# Supplementary figures and images for: RIPK2 induces docetaxel resistance in prostate cancer through the NF-κB/P-gp signaling pathway
Source: PLoS One. 2026 Jan 21;21(1):e0341445. doi: 10.1371/journal.pone.0341445 (PMC12822930; doi:10.1371/journal.pone.0341445)

Fig2B:

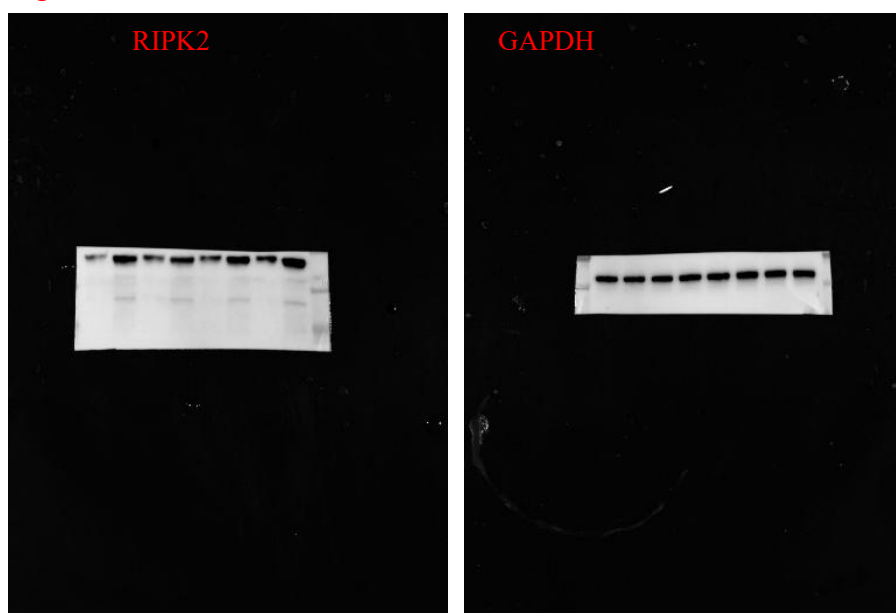

Fig2E:

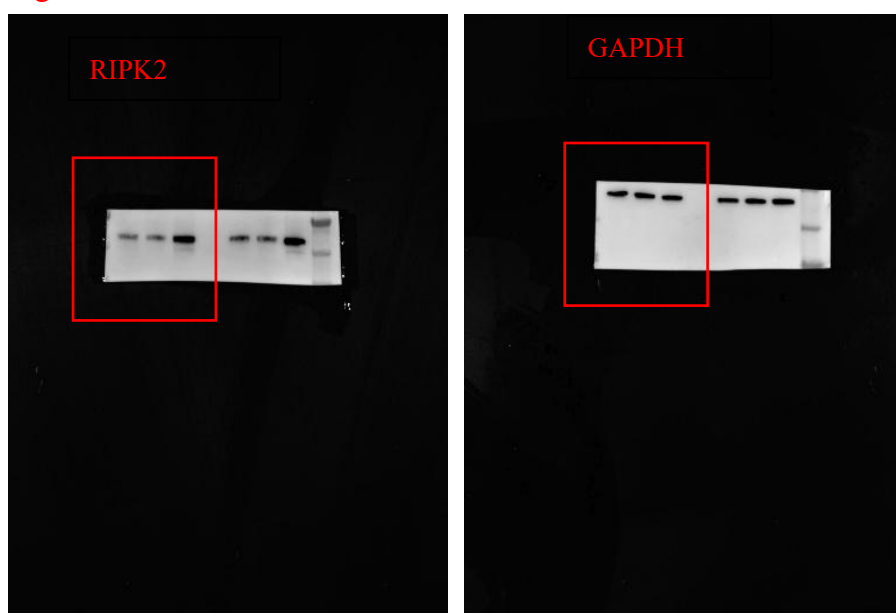

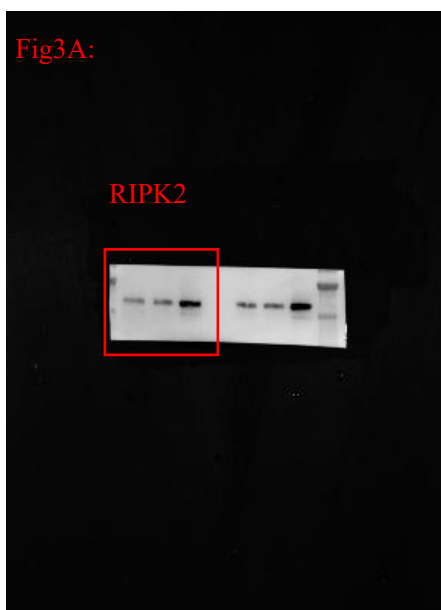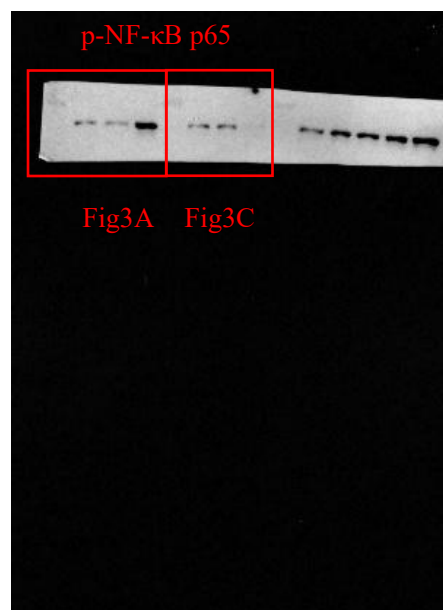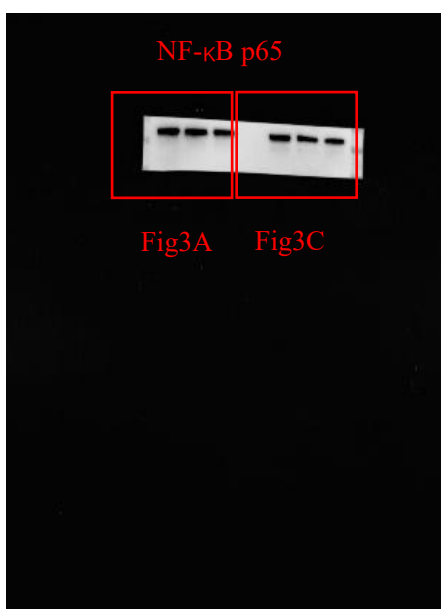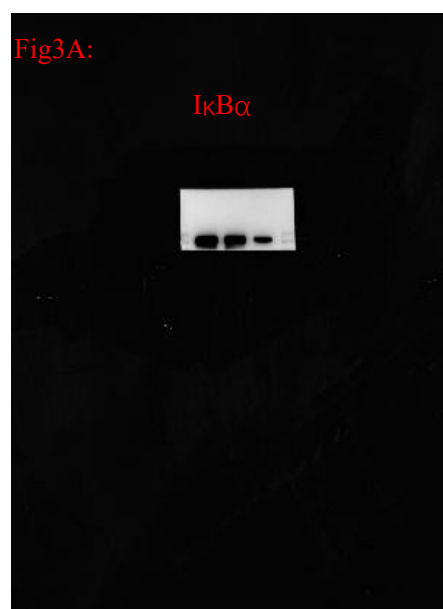

Fig3A:

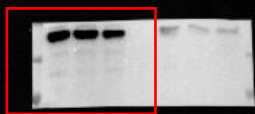

Fig3C:

RIPK2

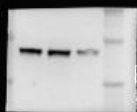

I $\kappa$ B $\alpha$

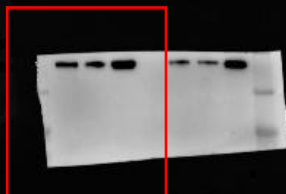

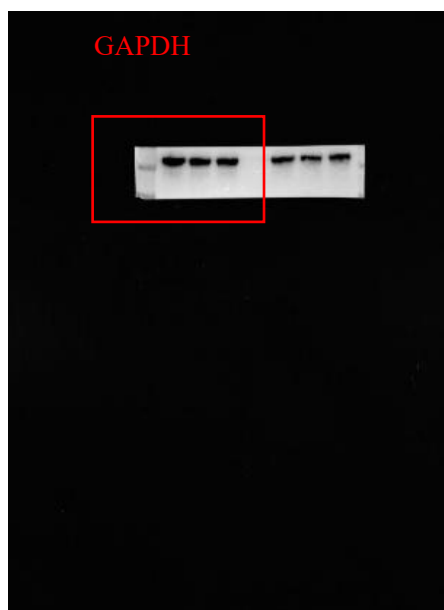

Fig4A:

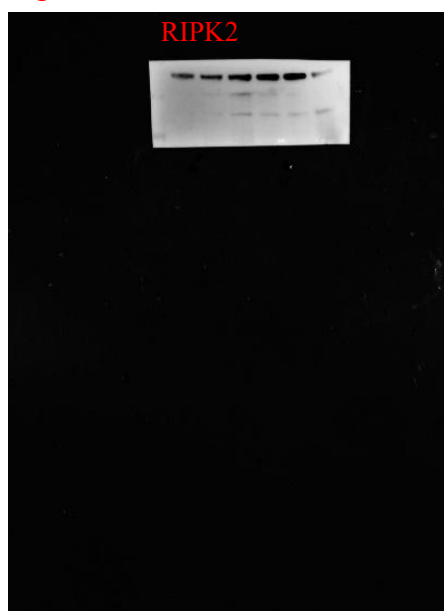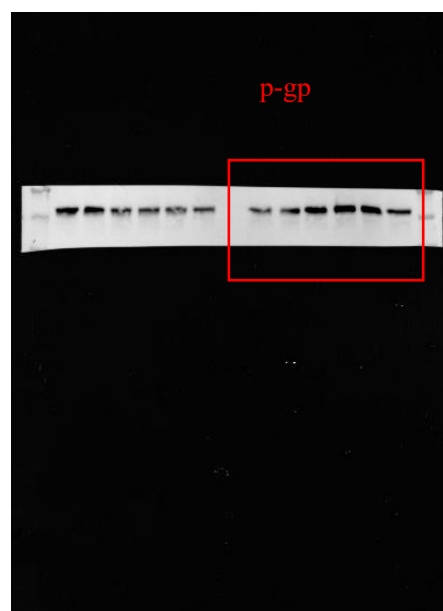

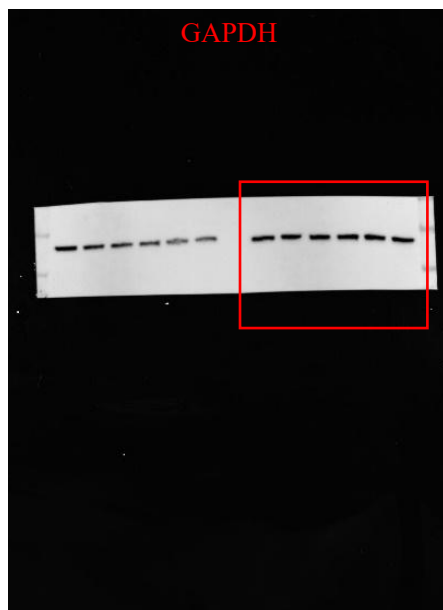

Fig4C:

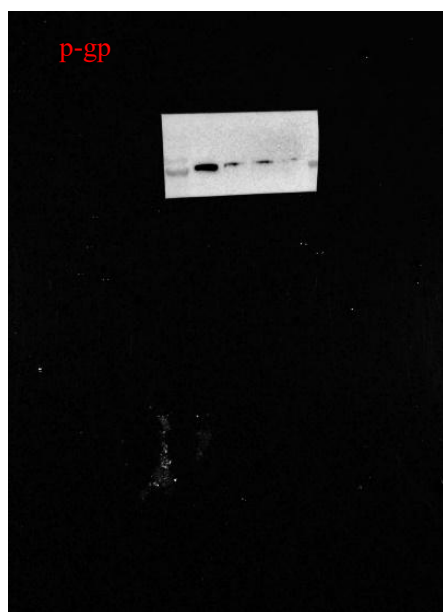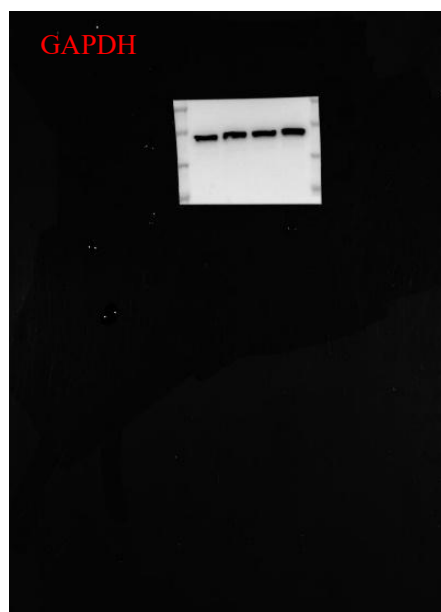

Fig4D:

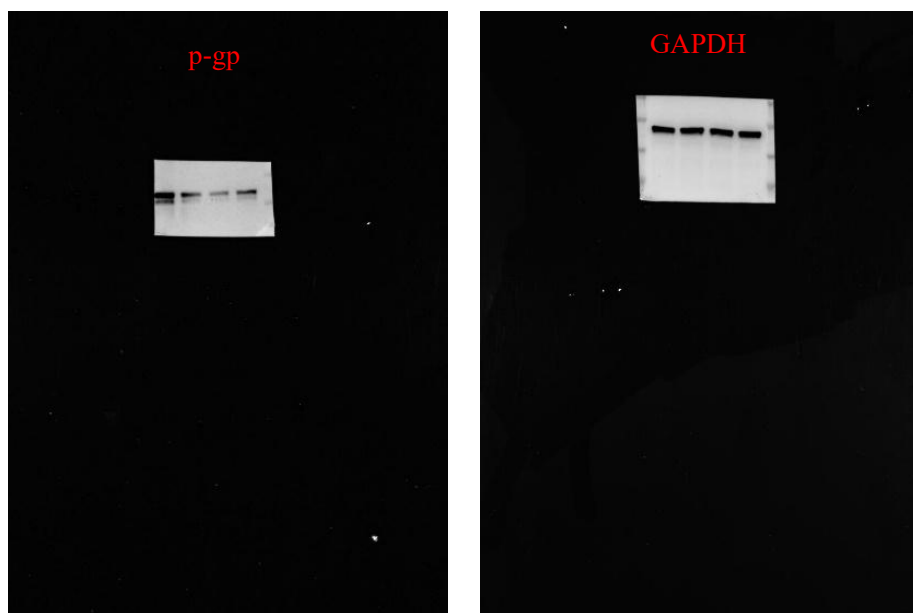

Supplement: S1 File — (PDF) [file pone.0341445.s001.pdf]
