# Supplementary material for: RIPK2 induces docetaxel resistance in prostate cancer through the NF-κB/P-gp signaling pathway
Source: PLoS One. 2026 Jan 21;21(1):e0341445. doi: 10.1371/journal.pone.0341445 (PMC12822930; doi:10.1371/journal.pone.0341445)
Supplement: S2 File — (PDF) [file pone.0341445.s002.pdf]

Fig2A:

| X | Group A | | | Group B | | |
|---|---|---|---|---|---|---|
| log[Agonist], M | 22RV1 | | | 22RV1/DTX | | |
| X | A:Y1 | A:Y2 | A:Y3 | B:Y1 | B:Y2 | B:Y3 |
| -3.000000 | 100.0 | 99.6 | 103.2 | | | |
| -2.000000 | 101.1 | 98.9 | 96.3 | 99.3 | 100.1 | 102.1 |
| -1.000000 | 88.2 | 85.6 | 92.7 | 99.3 | 96.5 | 100.2 |
| 0.000000 | 63.6 | 60.6 | 62.5 | 92.2 | 96.6 | 89.9 |
| 1.000000 | 40.6 | 42.1 | 33.2 | 64.2 | 66.7 | 68.3 |
| 1.698970 | 19.8 | 22.1 | 23.8 | 48.5 | 45.2 | 37.4 |
| 2.000000 | | | | 36.7 | 33.6 | 27.5 |

| X | Group A | | | Group B | | |
|---|---|---|---|---|---|---|
| log[Agonist], M | C4-2 | | | C4-2DTX | | |
| X | A:Y1 | A:Y2 | A:Y3 | B:Y1 | B:Y2 | B:Y3 |
| -3.000000 | 100.2 | 100.2 | 99.1 | | | |
| -2.000000 | 95.6 | 93.3 | 90.6 | 98.6 | 100.3 | 99.1 |
| -1.000000 | 87.6 | 85.2 | 89.9 | 89.9 | 88.3 | 95.6 |
| 0.000000 | 69.7 | 71.5 | 73.2 | 86.6 | 83.2 | 80.3 |
| 1.000000 | 46.3 | 51.3 | 47.2 | 68.8 | 64.4 | 61.8 |
| 1.698970 | 35.2 | 26.1 | 28.1 | 45.6 | 44.1 | 46.2 |
| 2.000000 | | | | 33.5 | 33.1 | 30.2 |

| X | Group A | | | Group B | | |
|---|---|---|---|---|---|---|
| log[Agonist], M | PC-3 | | | PC-3/DTX | | |
| X | A:Y1 | A:Y2 | A:Y3 | B:Y1 | B:Y2 | B:Y3 |
| -3.000000 | 100.0 | 101.3 | 97.7 | | | |
| -2.000000 | 90.0 | 95.2 | 91.8 | 99.7 | 97.3 | 100.5 |
| -1.000000 | 81.0 | 84.3 | 86.6 | 91.8 | 87.2 | 93.1 |
| 0.000000 | 61.0 | 66.6 | 67.4 | 80.0 | 79.7 | 73.2 |
| 1.000000 | 48.7 | 43.1 | 46.7 | 65.7 | 63.1 | 68.3 |
| 1.698970 | 31.0 | 29.2 | 34.1 | 46.8 | 50.6 | 52.1 |
| 2.000000 | | | | 32.2 | 34.7 | 29.8 |

| X | Group A | | | Group B | | |
|---|---|---|---|---|---|---|
| log[Agonist], M | DU145 | | | DU145/DTX | | |
| X | A:Y1 | A:Y2 | A:Y3 | B:Y1 | B:Y2 | B:Y3 |
| -3.000000 | 100.2 | 98.8 | 99.1 | | | |
| -2.000000 | 91.2 | 85.2 | 86.5 | 97.3 | 98.2 | 99.1 |
| -1.000000 | 84.6 | 82.6 | 79.6 | 89.9 | 88.3 | 90.6 |
| 0.000000 | 65.3 | 60.1 | 64.1 | 85.5 | 83.2 | 80.3 |
| 1.000000 | 46.3 | 43.6 | 47.6 | 63.3 | 60.6 | 62.8 |
| 1.698970 | 35.2 | 33.1 | 28.1 | 45.6 | 43.1 | 47.2 |
| 2.000000 | | | | 28.8 | 36.3 | 30.2 |

Fig2B:

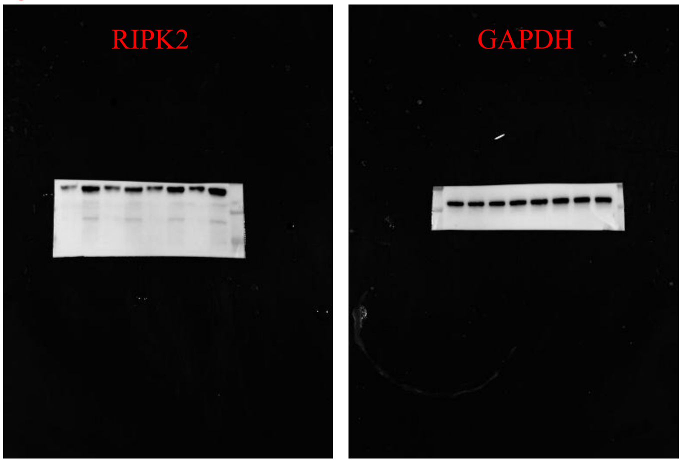


Fig 2C:

| | X | Group A | | | Group B | | | Group C | | |
|---|---|---|---|---|---|---|---|---|---|---|
| | log[Agonist], M | Control | | | siControl | | | siRIPK2 | | |
| | X | A:Y1 | A:Y2 | A:Y3 | B:Y1 | B:Y2 | B:Y3 | C:Y1 | C:Y2 | C:Y3 |
| 1 | -3.000000 | | | | | | | 98.2000 | 100.0 | 101.8 |
| 2 | -2.000000 | 99.3 | 100.1 | 102.1 | 97.6 | 98.2 | 102.3 | 101.1000 | 98.9 | 96.3 |
| 3 | -1.000000 | 99.3 | 96.5 | 100.2 | 98.7 | 95.8 | 99.1 | 94.2000 | 91.1 | 95.6 |
| 4 | 0.000000 | 92.2 | 93.6 | 89.9 | 97.3 | 90.8 | 90.5 | 79.1000 | 77.8 | 73.9 |
| 5 | 1.000000 | 64.2 | 66.7 | 70.3 | 70.3 | 64.4 | 67.2 | 49.5000 | 56.2 | 45.5 |
| 6 | 1.698970 | 48.5 | 45.2 | 37.4 | 46.3 | 44.1 | 35.2 | 31.6000 | 30.3 | 36.7 |
| 7 | 2.000000 | 36.7 | 33.6 | 27.5 | 33.9 | 34.1 | 26.8 | | | |

| | X | Group A | | | Group B | | | Group C | | |
|---|---|---|---|---|---|---|---|---|---|---|
| | log[Agonist], M | Control | | | siControl | | | siRIPK2 | | |
| | X | A:Y1 | A:Y2 | A:Y3 | B:Y1 | B:Y2 | B:Y3 | C:Y1 | C:Y2 | C:Y3 |
| 1 | -3.000000 | | | | | | | 100.2000 | 100.2 | 99.1 |
| 2 | -2.000000 | 98.6 | 100.3 | 99.1 | 100.0 | 100.3 | 96.7 | 95.6000 | 93.3 | 90.6 |
| 3 | -1.000000 | 89.9 | 88.3 | 95.6 | 91.2 | 89.9 | 97.2 | 87.6000 | 85.2 | 89.9 |
| 4 | 0.000000 | 86.6 | 91.2 | 84.3 | 91.3 | 81.4 | 87.3 | 76.7000 | 79.5 | 73.2 |
| 5 | 1.000000 | 68.8 | 64.4 | 61.8 | 64.3 | 65.0 | 63.7 | 56.3000 | 55.3 | 47.2 |
| 6 | 1.698970 | 45.6 | 44.1 | 46.2 | 46.6 | 43.2 | 45.1 | 35.2000 | 27.8 | 30.3 |
| 7 | 2.000000 | 33.5 | 33.1 | 30.2 | 30.7 | 32.6 | 33.8 | | | |

| | X | Group A | | | Group B | | | Group C | | |
|---|---|---|---|---|---|---|---|---|---|---|
| | log[Agonist], M | Control | | | siControl | | | siRIPK2 | | |
| | X | A:Y1 | A:Y2 | A:Y3 | B:Y1 | B:Y2 | B:Y3 | C:Y1 | C:Y2 | C:Y3 |
| 1 | -3.000000 | | | | | | | 98.1 | 102.1 | 97.3 |
| 2 | -2.000000 | 101.30000 | 99.2 | 98.9 | 96.5 | 101.2 | 102.3 | 88.2 | 92.1 | 93.1 |
| 3 | -1.000000 | 90.60000 | 88.7 | 96.0 | 91.8 | 89.3 | 95.1 | 83.6 | 88.7 | 87.1 |
| 4 | 0.000000 | 86.70000 | 80.2 | 77.1 | 83.7 | 83.6 | 80.3 | 69.3 | 73.6 | 71.2 |
| 5 | 1.000000 | 64.30000 | 65.5 | 69.2 | 69.5 | 60.1 | 66.6 | 49.2 | 43.3 | 47.6 |
| 6 | 1.698970 | 45.10000 | 50.1 | 53.7 | 45.1 | 49.8 | 54.5 | 36.5 | 32.2 | 34.6 |
| 7 | 2.000000 | 36.50000 | 34.8 | 28.6 | 30.2 | 32.3 | 32.1 | | | |

| X | Group A | | | Group B | | | Group C | | |
|---|---|---|---|---|---|---|---|---|---|
| log[Agonist], M | Control | | | siControl | | | siRIPK2 | | |
| X | A:Y1 | A:Y2 | A:Y3 | B:Y1 | B:Y2 | B:Y3 | C:Y1 | C:Y2 | C:Y3 |
| 1 -3.000000 | | | | | | | 102.2 | 97.3 | 101.6 |
| 2 -2.000000 | 97.3 | 100.5 | 99.1 | 99.6 | 99.2 | 99.8 | 95.2 | 94.6 | 89.5 |
| 3 -1.000000 | 89.9 | 88.3 | 94.6 | 86.5 | 94.2 | 90.6 | 86.6 | 88.1 | 93.2 |
| 4 0.000000 | 85.5 | 87.2 | 90.6 | 88.6 | 85.3 | 85.1 | 80.6 | 82.5 | 77.8 |
| 5 1.000000 | 65.3 | 65.5 | 62.8 | 64.5 | 63.8 | 61.1 | 50.3 | 47.6 | 56.3 |
| 6 1.698970 | 45.6 | 43.1 | 44.2 | 46.3 | 41.2 | 48.6 | 32.2 | 36.1 | 35.1 |
| 7 2.000000 | 28.8 | 36.3 | 30.2 | 30.6 | 34.3 | 30.1 | | | |

Fig 2D:

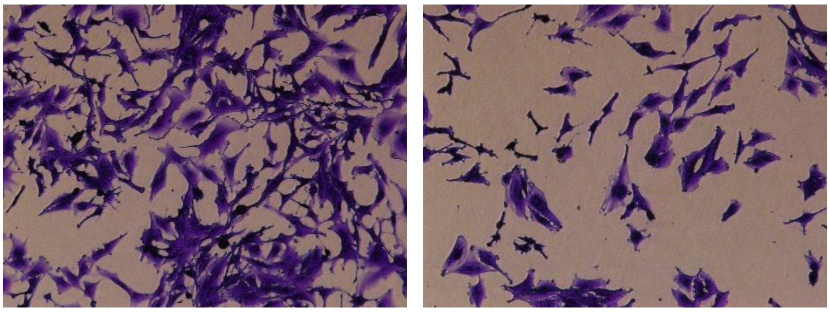

Fig2E:

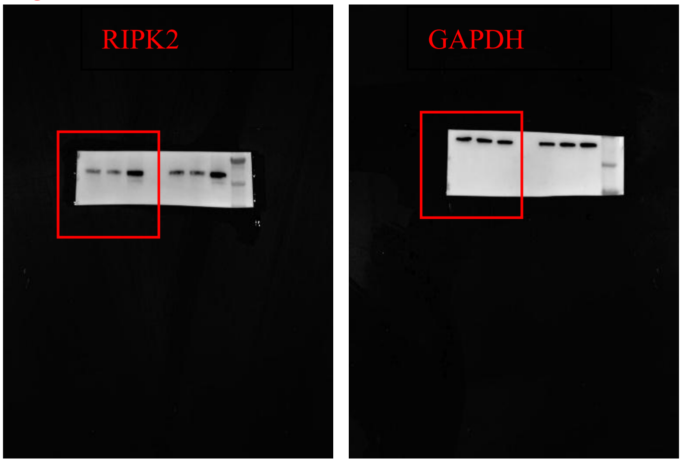


Fig 2F:

| X | Group A | | | Group B | | | Group C | | |
|---|---|---|---|---|---|---|---|---|---|
| log[Agonist], M | Control | | | Vector | | | RIPK2 plasmid | | |
| X | A:Y1 | A:Y2 | A:Y3 | B:Y1 | B:Y2 | B:Y3 | C:Y1 | C:Y2 | C:Y3 |
| 1 -3.000000 | 100.0 | 98.6 | 101.4 | 100.0 | 100.3 | 99.7 | | | |
| 2 -2.000000 | 90.3 | 96.7 | 93.5 | 90.6 | 95.2 | 91.7 | 99.7 | 97.3 | 100.5 |
| 3 -1.000000 | 82.2 | 85.6 | 87.7 | 83.5 | 82.3 | 86.6 | 91.8 | 96.3 | 93.1 |
| 4 0.000000 | 65.2 | 71.1 | 69.4 | 63.7 | 69.3 | 67.7 | 84.2 | 90.6 | 78.1 |
| 5 1.000000 | 46.5 | 42.1 | 43.3 | 44.2 | 43.1 | 41.9 | 60.2 | 56.3 | 65.7 |
| 6 1.698970 | 30.3 | 27.9 | 35.8 | 31.6 | 28.3 | 36.4 | 42.2 | 45.9 | 47.1 |
| 7 2.000000 | | | | | | | 29.1 | 31.0 | 25.8 |

Fig 2G:

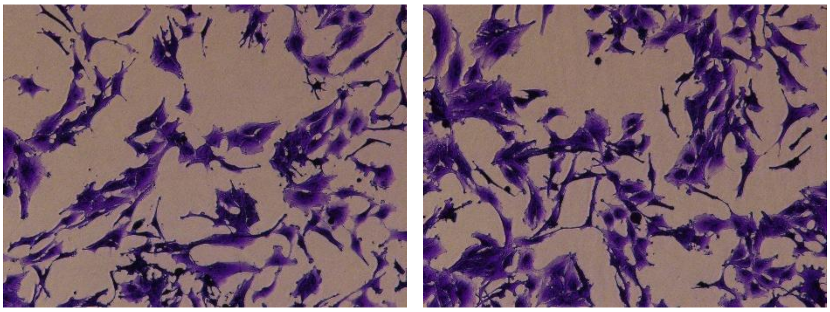

Fig3:

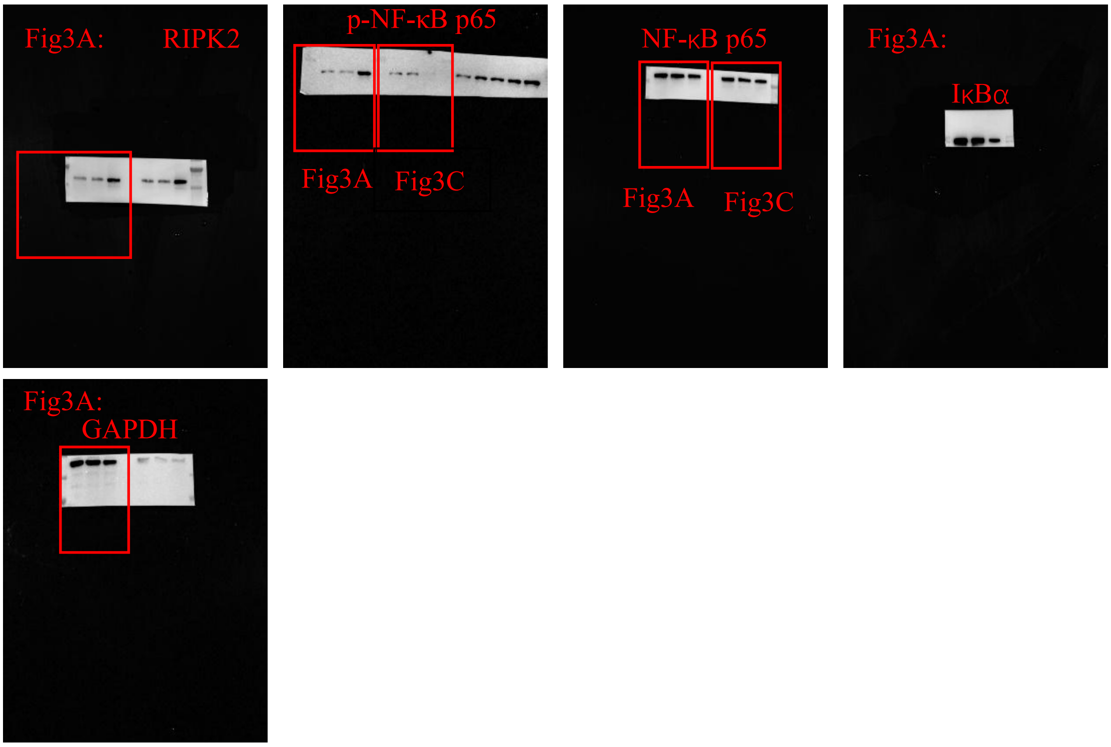


Fig3B:

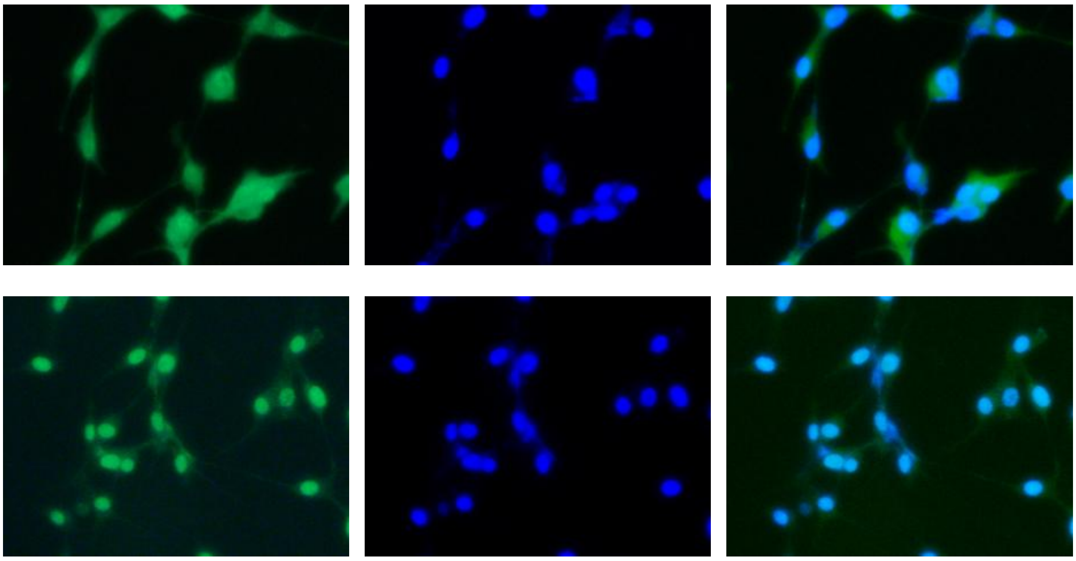

Fig3C:

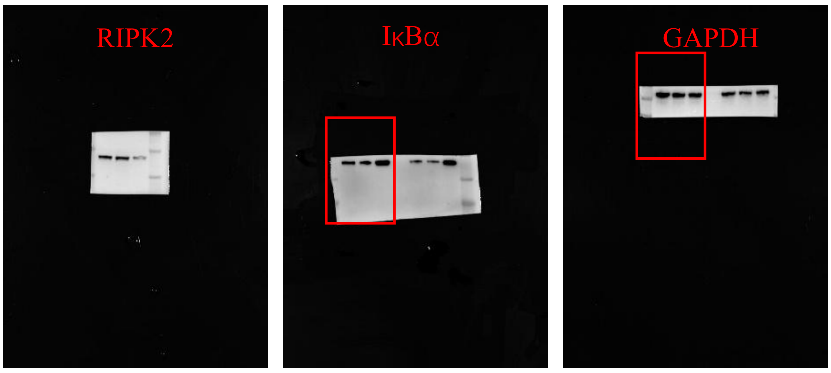


Fig3D:

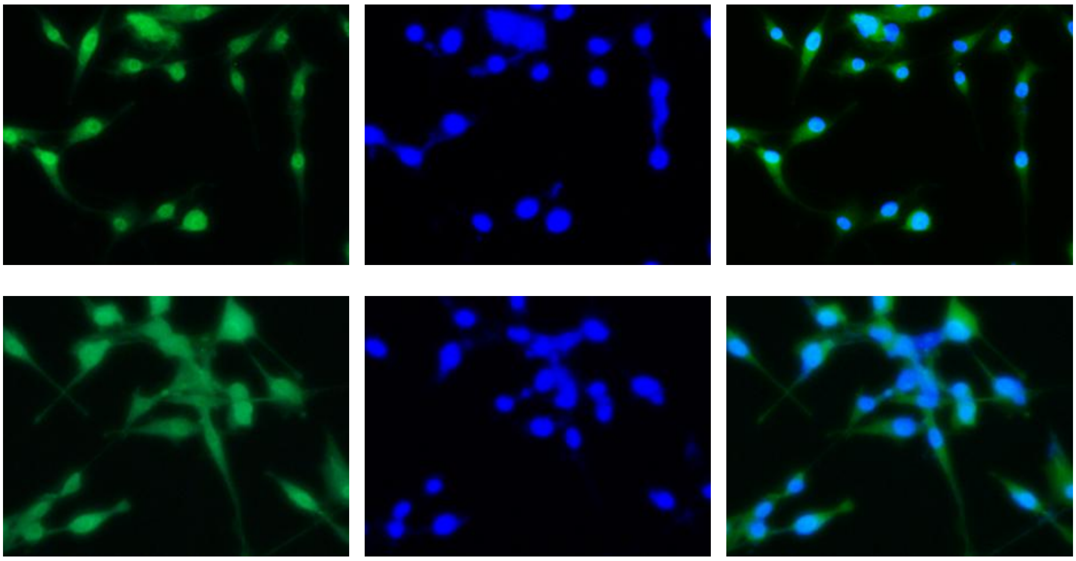

Fig4A:

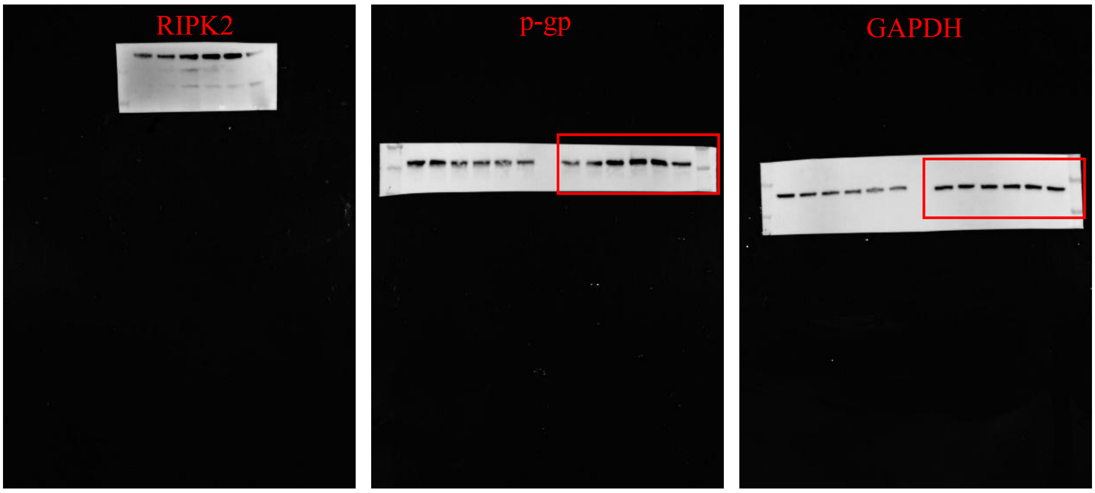

Fig4B:

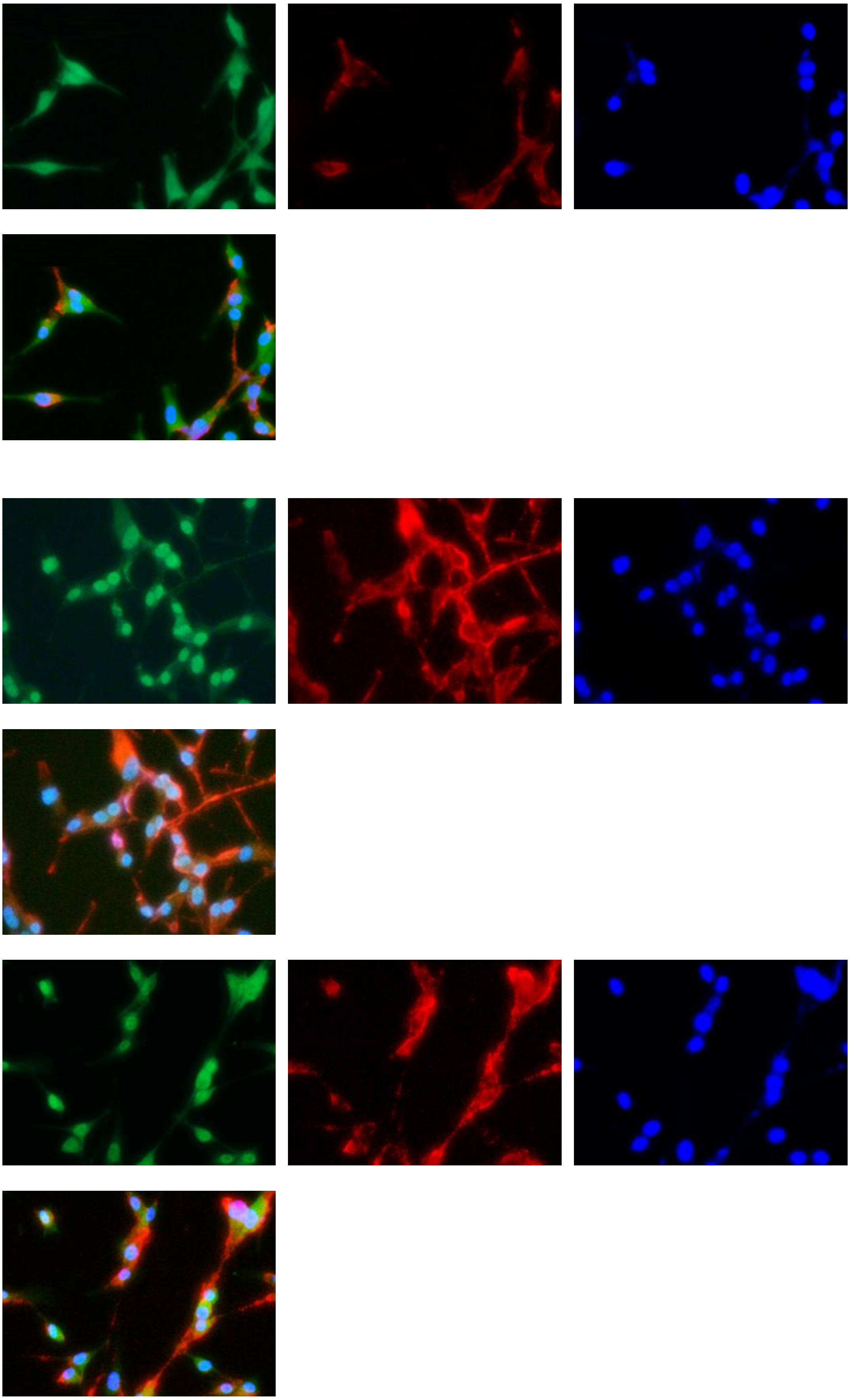

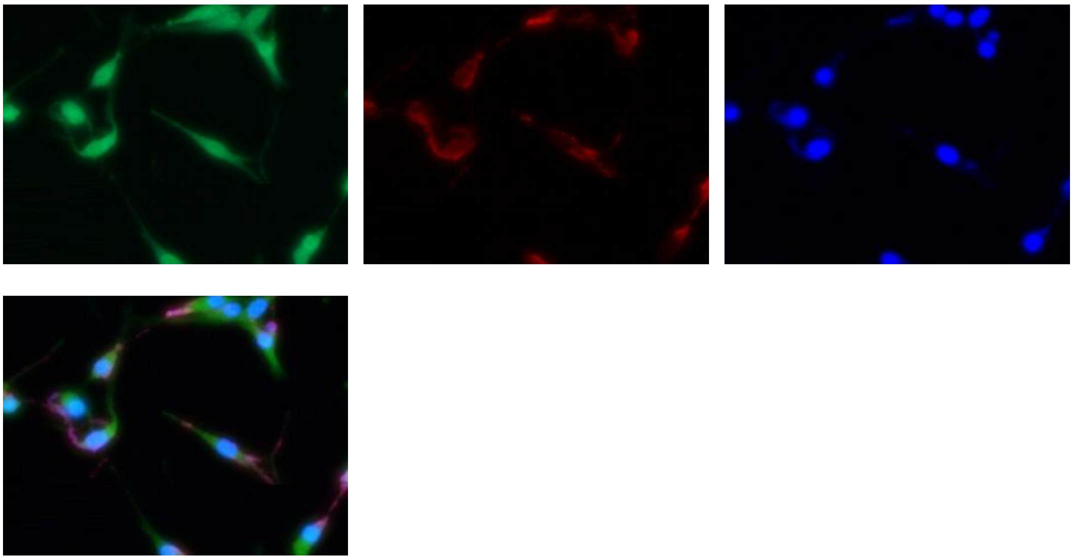

Fig4C:

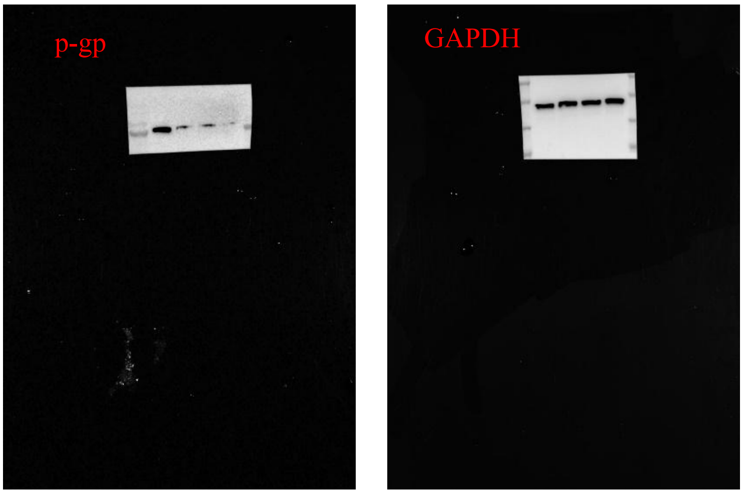


Fig4D:

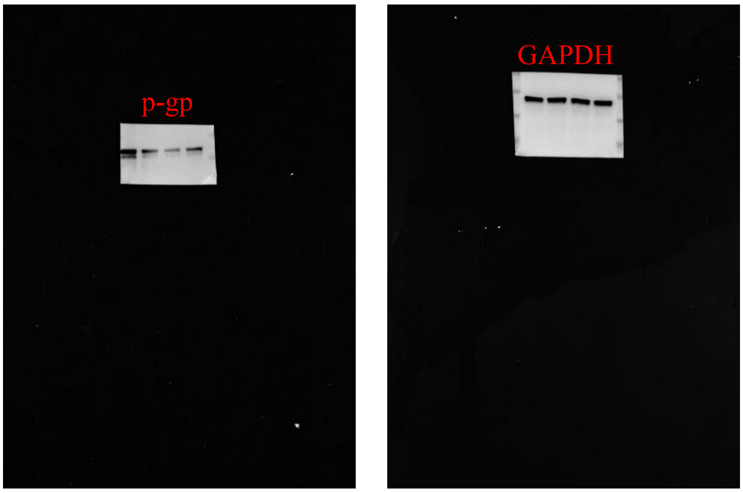

Fig5A:

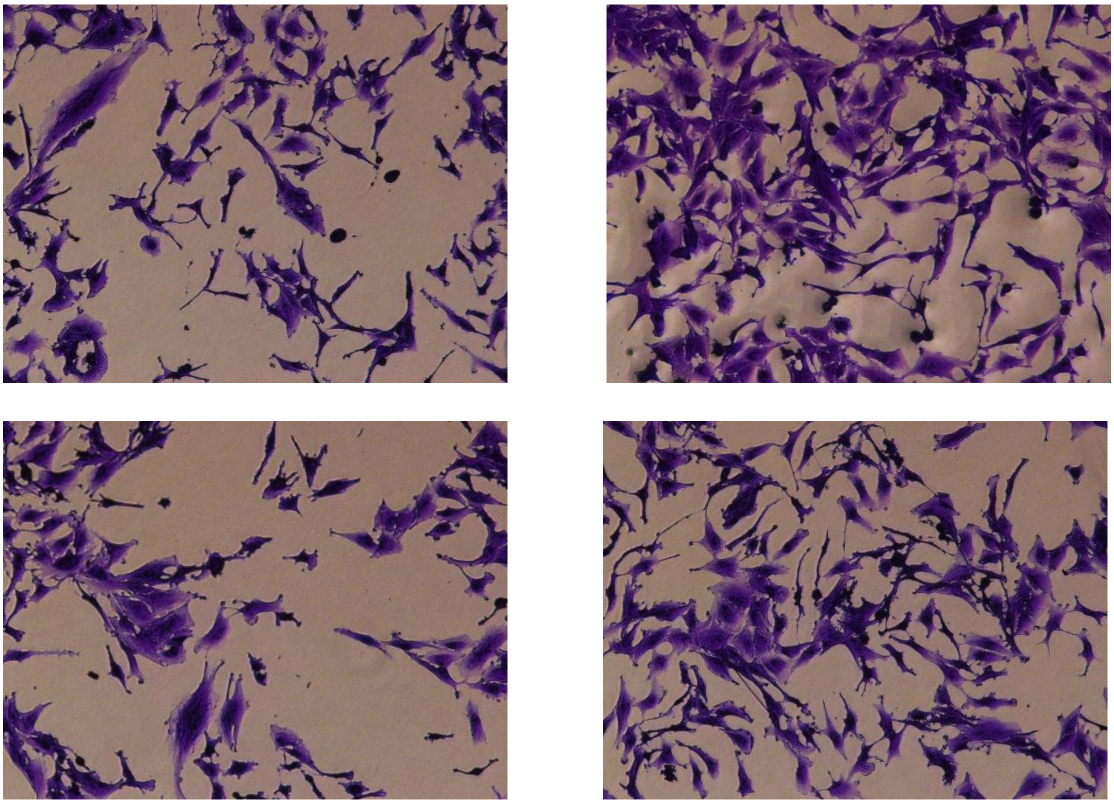

Fig5B:

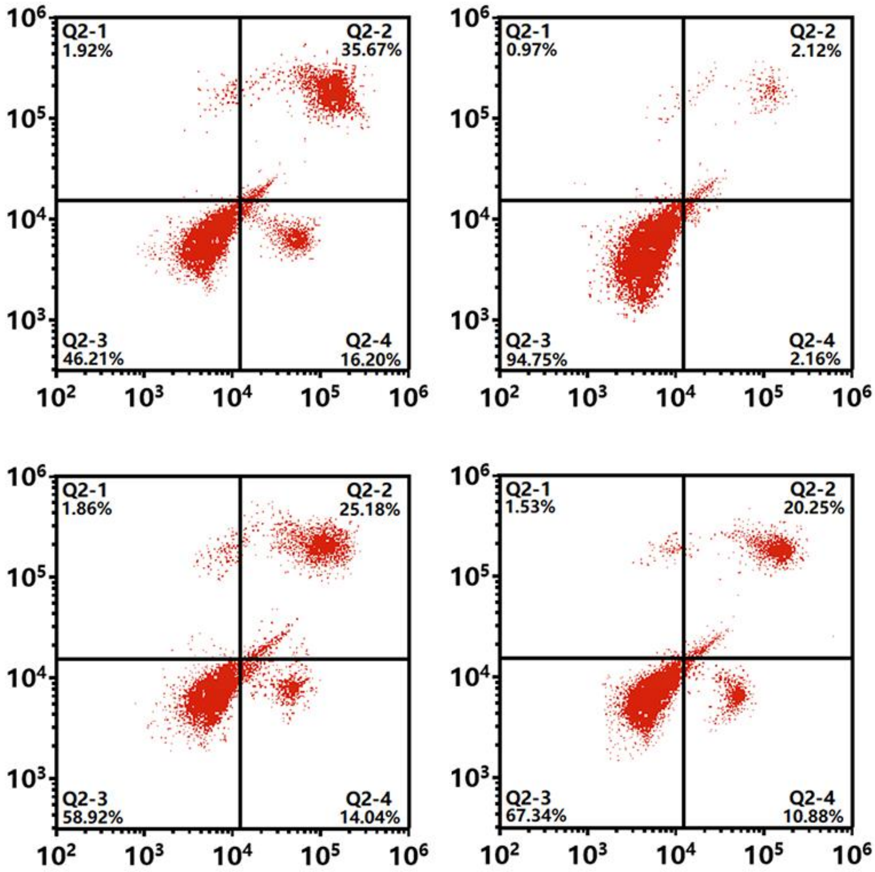

Q2-1
1.92%
Q2-2
35.67%
Q2-3
46.21%
Q2-4
16.20%
Q2-1
0.97%
Q2-2
2.12%
Q2-3
94.75%
Q2-4
2.16%
Q2-1
1.86%
Q2-2
25.18%
Q2-3
58.92%
Q2-4
14.04%
Q2-1
1.53%
Q2-2
20.25%
Q2-3
67.34%
Q2-4
10.88%

Fig5C:

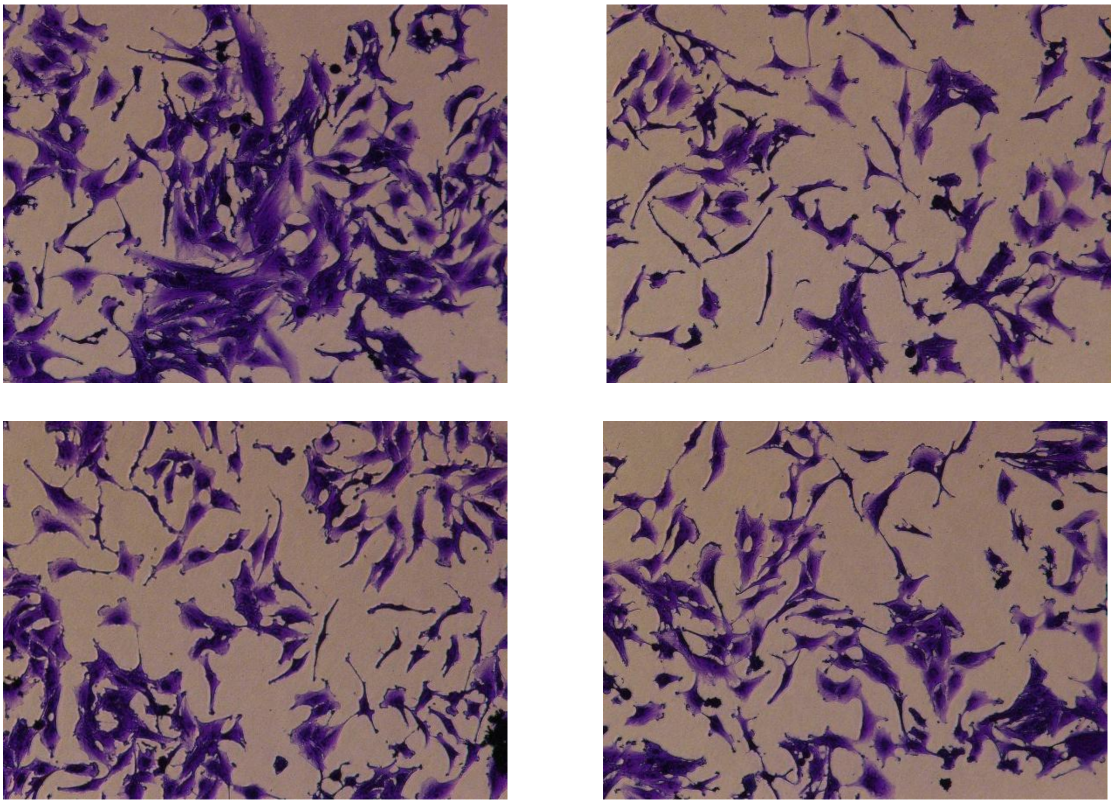

Fig5D:

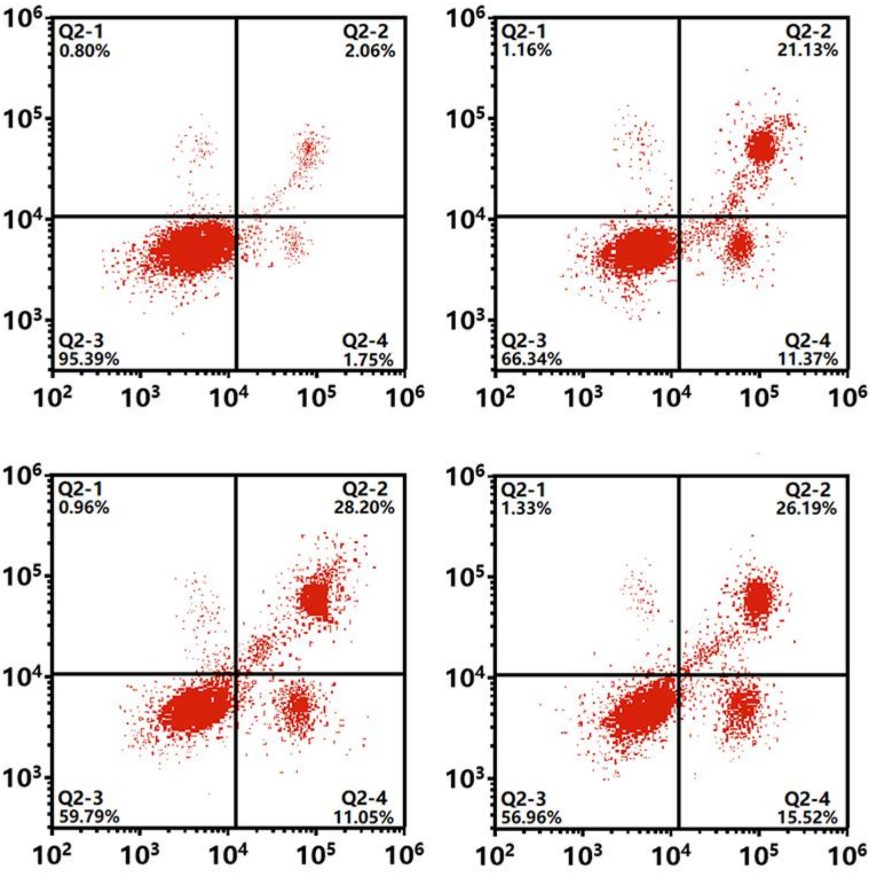
Q2-1
0.80%
Q2-2
2.06%
Q2-3
95.39%
Q2-4
1.75%
Q2-1
1.16%
Q2-2
21.13%
Q2-3
66.34%
Q2-4
11.37%
Q2-1
0.96%
Q2-2
28.20%
Q2-3
59.79%
Q2-4
11.05%
Q2-1
1.33%
Q2-2
26.19%
Q2-3
56.96%
Q2-4
15.52%
$10^2$
$10^3$
$10^4$
$10^5$
$10^6$

Fig6A:

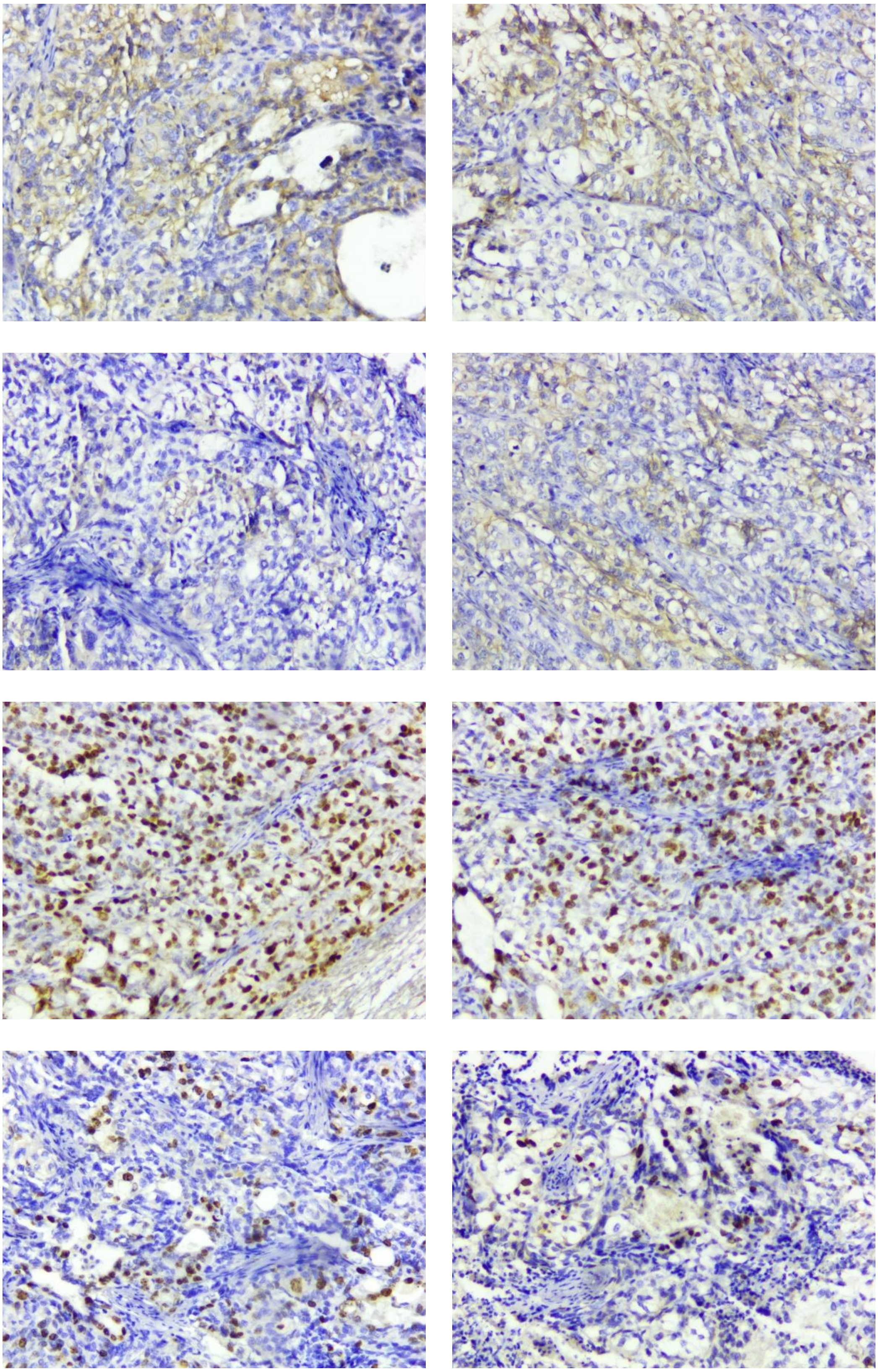

Fig6B:

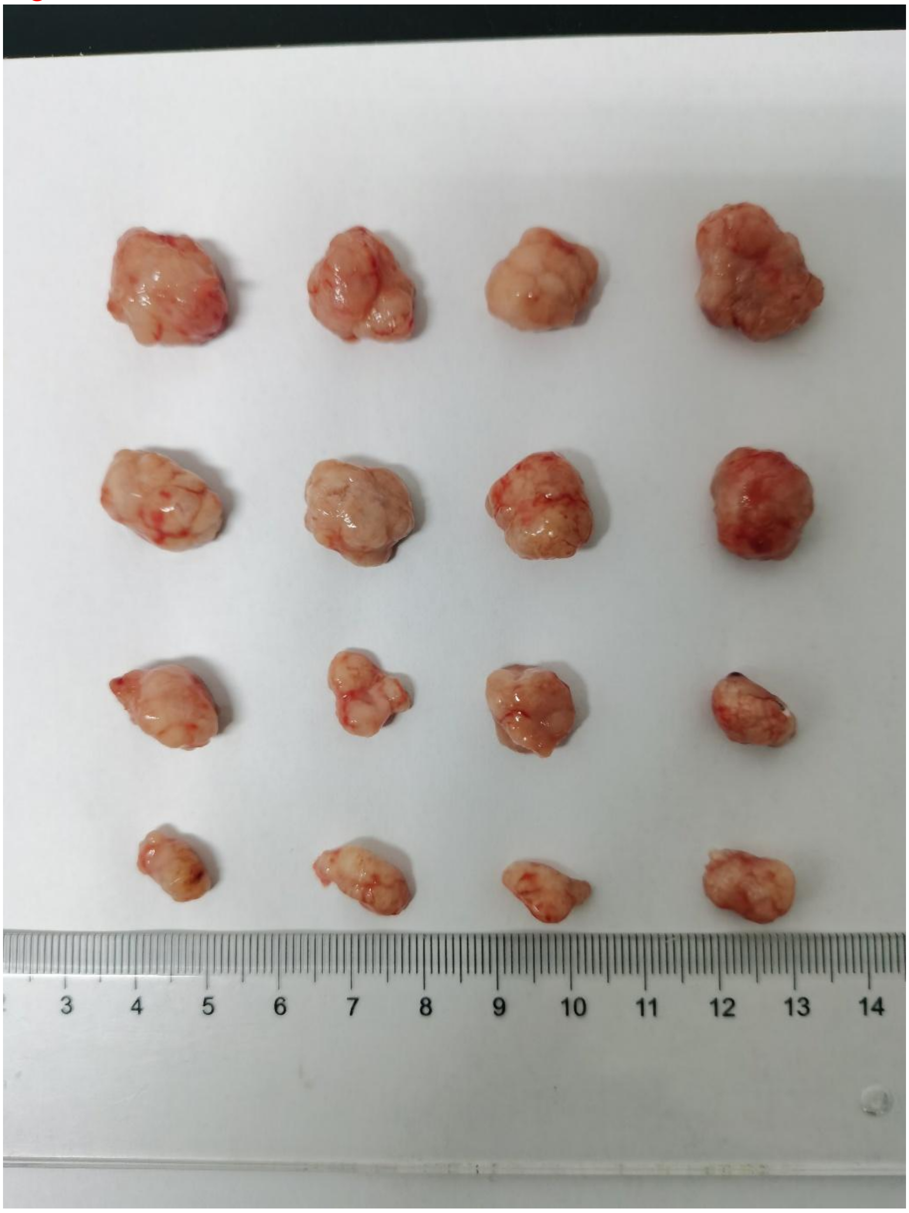


Fig6C:

| Table format: Grouped | | Group A Control | | | Group B DTX | | | Group C DTX+GSK583 | | | Group D DTX+Tariquidar | | |
|---|---|---|---|---|---|---|---|---|---|---|---|---|---|
| | | Mean | SD | N | Mean | SD | N | Mean | SD | N | Mean | SD | N |
| 1 | 1 | 205.7890 | 38.9 | 4 | 204.6230 | 40.15 | 4 | 203.8790 | 41.37 | 4 | 203.5070 | 42.800 | 4 |
| 2 | 3 | 243.5000 | 43.6 | 4 | 240.7580 | 40.86 | 4 | 239.2000 | 43.59 | 4 | 237.3000 | 42.874 | 4 |
| 3 | 5 | 303.4595 | 55.3 | 4 | 299.6055 | 53.17 | 4 | 268.3907 | 50.16 | 4 | 262.6000 | 49.865 | 4 |
| 4 | 8 | 380.9048 | 56.7 | 4 | 377.6894 | 62.14 | 4 | 303.1776 | 58.89 | 4 | 293.2895 | 56.632 | 4 |
| 5 | 10 | 472.9064 | 83.1 | 4 | 461.8804 | 86.89 | 4 | 342.4328 | 60.78 | 4 | 339.6099 | 61.178 | 4 |
| 6 | 12 | 567.3535 | 93.6 | 4 | 572.8814 | 98.89 | 4 | 385.8000 | 62.26 | 4 | 364.1058 | 58.874 | 4 |
| 7 | 15 | 667.4780 | 113.2 | 4 | 632.6727 | 123.56 | 4 | 442.2000 | 76.53 | 4 | 408.9200 | 65.260 | 4 |
| 8 | 17 | 850.9708 | 166.8 | 4 | 802.5020 | 173.58 | 4 | 500.1754 | 81.12 | 4 | 426.5453 | 71.120 | 4 |
| 9 | 19 | 1073.6785 | 202.3 | 4 | 904.7856 | 198.89 | 4 | 561.5890 | 89.96 | 4 | 442.5710 | 73.580 | 4 |
| 10 | 21 | 1185.6350 | 223.6 | 4 | 1103.5690 | 202.78 | 4 | 605.5890 | 93.37 | 4 | 462.5710 | 80.170 | 4 |
| 11 | 23 | 1335.6350 | 243.8 | 4 | 1268.7562 | 232.58 | 4 | 645.7854 | 95.87 | 4 | 487.8523 | 82.260 | 4 |
| 12 | 25 | 1605.6350 | 301.2 | 4 | 1532.6000 | 300.70 | 4 | 678.6547 | 101.23 | 4 | 501.1235 | 86.360 | 4 |
| 13 | 28 | 1783.0000 | 338.6 | 4 | 1683.6425 | 308.60 | 4 | 708.7589 | 120.45 | 4 | 510.7895 | 90.120 | 4 |

Fig6D:

| Table format: Grouped | | Group A Control | | | | Group B DTX | | | | Group C DTX+GSK583 | | | | Group D DTX+Tariquidar | | | |
|---|---|---|---|---|---|---|---|---|---|---|---|---|---|---|---|---|---|
| | | A:1 | A:2 | A:3 | A:4 | B:1 | B:2 | B:3 | B:4 | C:1 | C:2 | C:3 | C:4 | D:1 | D:2 | D:3 | D:4 |
| 1 | Title | 1.106 | 1.321 | 0.875 | 0.926 | 1.034 | 1.123 | 0.865 | 0.881 | 0.425 | 0.513 | 0.364 | 0.531 | 0.382 | 0.341 | 0.437 | 0.403 |
