# Supplementary material for: RIPK2 induces docetaxel resistance in prostate cancer through the NF-κB/P-gp signaling pathway
Source: PLoS One. 2026 Jan 21;21(1):e0341445. doi: 10.1371/journal.pone.0341445 (PMC12822930; doi:10.1371/journal.pone.0341445)
Supplement: Attachment — Submitted filename: Response to Reviewers.docx [file pone.0341445.s003.docx]

2025/11/05

Dear Editor and Reviewers:

Thank you very much for your time and constructive comments on our manuscript entitled " RIPK2 induces docetaxel resistance in prostate cancer through the NF-κB/P-gp signaling pathway". We greatly appreciate the valuable suggestions, which have helped us significantly improve the quality of this work. According to the reviewers' comments, we have carefully revised the manuscript and addressed all the concerns raised. The main revisions are summarized as follows, and detailed responses to each comment are provided below. We hope the revised version meets the publication standards of *PLOS ONE*.

Sincerely,

Qian Liu

Department of Urology, First Central Clinical College, Tianjin Medical University, No. 24, Fukang Road, Nankai District, Tianjin, 300190, China. +86 18963106651

[simonlq1971@126.com](mailto:simonlq1971@126.com)

Journal Requirements:

1. Please ensure that your manuscript meets PLOS ONE's style requirements, including those for file naming. The PLOS ONE style templates can be found at

https://journals.plos.org/plosone/s/file?id=wjVg/PLOSOne_formatting_sample_main_body.pdf and

https://journals.plos.org/plosone/s/file?id=ba62/PLOSOne_formatting_sample_title_authors_affiliations.pdf

Reply: We have reviewed the manuscript according to the formatting requirements and file naming conventions specified by PLOS ONE.

2. Thank you for stating the following financial disclosure:

“This work was supported by the Tianjin Key Medical Discipline(Specialty) Construction Project.”

Please state what role the funders took in the study. If the funders had no role, please state: "The funders had no role in study design, data collection and analysis, decision to publish, or preparation of the manuscript."

If this statement is not correct you must amend it as needed.

Please include this amended Role of Funder statement in your cover letter; we will change the online submission form on your behalf.

Reply: We have incorporated the revised statement of the funding agency's role into the cover letter.

3. PLOS ONE now requires that authors provide the original uncropped and unadjusted images underlying all blot or gel results reported in a submission’s figures or Supporting Information files. This policy and the journal’s other requirements for blot/gel reporting and figure preparation are described in detail at https://journals.plos.org/plosone/s/figures#loc-blot-and-gel-reporting-requirements and https://journals.plos.org/plosone/s/figures#loc-preparing-figures-from-image-files. When you submit your revised manuscript, please ensure that your figures adhere fully to these guidelines and provide the original underlying images for all blot or gel data reported in your submission. See the following link for instructions on providing the original image data: https://journals.plos.org/plosone/s/figures#loc-original-images-for-blots-and-gels.

In your cover letter, please note whether your blot/gel image data are in Supporting Information or posted at a public data repository, provide the repository URL if relevant, and provide specific details as to which raw blot/gel images, if any, are not available. Email us at plosone@plos.org if you have any questions.

Reply: The blot/gel image data are included in the Supplementary Materials. We have supplemented the relevant information in the cover letter.

Additional Editor Comments:

The manuscript investigates the role of RIPK2 in mediating docetaxel (DTX) resistance in prostate cancer, proposing a mechanism via NF-kB activation and upregulation of P-glycoprotein (P-gp). The study includes in vitro experiments, TCGA dataset analysis, and xenograft models. The topic is relevant to therapeutic resistance in prostate cancer and fits PLOS ONE's emphasis on scientific rigour. However, several clarifications and improvements are needed before the manuscript can be considered for publication.

1. TCGA/UALCAN analysis- clarification needed:

The use of the UALCAN portal for TCGA analysis is appropriate, but a few reporting details would improve transparency and reproducibility. In particular, please indicate: How many tumour and normal samples were included? What statistical test did UALCAN apply, and how were the Gleason score groups defined?

If default UALCAN settings were used, a brief statement to that effect would be sufficient.

Reply: We have incorporated the number of cases used in the TCGA/UALCAN analysis into the ‘Materials and Methods’ section. Additionally, we performed Gleason score grouping and statistical analysis using default settings, with supplementary descriptions provided in the manuscript.

2. Please indicate whether in vitro data (e.g., IC50) were based on biological triplicates, and how many replicates were performed.

Reply: In vitro data (e.g., IC50 values) were based on biological triplicate experiments, with each biological replicate performed in at least three technical repeats to ensure reproducibility. We have supplemented the relevant descriptions in the manuscript.

3. The in vivo experiments are generally well described, but to meet PLOS ONE and ARRIVE reporting standards, a few brief clarifications would improve transparency and reproducibility.

Please specify the number of animals per group in the Methods

Indicate whether randomisation was used when assigning mice to treatment groups.

State whether blinding was applied during tumour measurement or analysis (or clarify if not performed).

Briefly note the monitoring frequency and any humane endpoint criteria used before the scheduled sacrifice.

clarify the nature of the control treatment/vehicle given to the drug-control group.

These additions would align the manuscript with ARRIVE expectations without requiring new experiments.

Reply: We have supplemented the relevant descriptions in the manuscript.

4. Writing and Style: Minor language edits would improve clarity and consistency. There are occasional tense shifts and missing articles. The introduction is also quite long and could be streamlined to avoid repetition. A light language edit would help polish the text.

Reply: We have invited native English speakers with academic writing experience to polish the manuscript, focusing on optimizing language fluency, correcting grammatical errors, and refining academic expression to meet the journal’s standards.

Reviewers' comments:

5. Review Comments to the Author

Reviewer #1: I am writing to provide feedback on the manuscript titled " RIPK2 induces docetaxel resistance in prostate cancer through the NF-κB/P-gp signaling pathway " (PONE-D-25-24339) which I understand is under consideration for publication. After a thorough review, I have identified several areas that could benefit from further attention to enhance the overall quality and impact of the paper.

References and Citations: It is crucial to ensure uniformity in the formatting of all references and citations, adhering to the required style guide. Consistency in this aspect upholds the manuscript's professionalism.

Reply: We have verified the references and citations.

Clarity and Conciseness: The manuscript would benefit from a focus on clear and concise communication. Avoiding excessive jargon and simplifying complex ideas will make the paper more accessible to a broader audience.

Reply: We have invited native English speakers with academic writing experience to polish the manuscript, focusing on optimizing language fluency, correcting grammatical errors, and refining academic expression to meet the journal’s standards.

Data Presentation: I recommend a review of how figures and tables are integrated into the paper. They should be clearly labeled and directly referenced in the text, ensuring they effectively support the paper's conclusions.

Reply: We have systematically reviewed the manuscript data to identify and eliminate potential errors, including data entry inconsistencies, statistical calculation discrepancies, and figure caption mismatches.

Logical Flow: Ensuring a coherent flow throughout the paper, with seamless transitions between sections, is key to keeping the reader engaged and ensuring the paper is easily understandable.

Reply: We have reviewed and checked the entire text.

Abstract and Conclusion: These sections should succinctly summarize the main findings and their significance. It is important to avoid overstating results or generalizations not supported by the data.

Reply: We have reviewed and confirmed the abstract and conclusions.

Consistency in Terminology: Uniform use of technical terms throughout the manuscript is essential for clarity and to avoid potential confusion among readers.

Reply: We have reviewed and checked the entire text.

Objective Language: Maintaining an objective tone throughout the paper is crucial. Subjective or evaluative language should be reserved for discussing hypotheses or theoretical frameworks.

Reply: We have reviewed and checked the entire text.

Compliance with Journal Guidelines: Finally, ensuring that the manuscript aligns with the journal's specific guidelines in terms of formatting, submission, and ethics will facilitate a smoother review and publication process.

Reply: We have checked the manuscript against the journal guidelines.

I trust these suggestions will be valuable in enhancing the manuscript's effectiveness and suitability for publication. Thank you for the opportunity to review this work.
