# Supplementary material for: RIPK2 induces docetaxel resistance in prostate cancer through the NF-κB/P-gp signaling pathway
Source: PLoS One. 2026 Jan 21;21(1):e0341445. doi: 10.1371/journal.pone.0341445 (PMC12822930; doi:10.1371/journal.pone.0341445)
Supplement: Attachment — Submitted filename: Response_to_Reviewers_auresp_2.docx [file pone.0341445.s004.docx]

2025/12/11

Dear Editor:

We thank the editors and reviewers for all their efforts in reviewing the manuscript. In response to the comments raised in the second round of review, we hereby provide detailed data regarding the maximum tumor size in our xenograft experiments. None of the xenograft tumors exceeded 10% of the host mice’s body weight, which is in strict compliance with the ethical guidelines for animal experiments. The largest tumor, with a volume of 1991.23 mm³ and a weight of 1.321 g, accounted for only 4.98% of the corresponding mouse’s body weight (26.5 g). It should be noted that tumor volume measurements might be slightly overestimated due to the irregular morphology of xenograft tumors; however, the overall results are consistent with our experimental expectations.

Thank you for your consideration. I look forward to hearing from you.

Sincerely,

Qian Liu

Department of Urology, First Central Clinical College, Tianjin Medical University, No. 24, Fukang Road, Nankai District, Tianjin, 300190, China. +86 18963106651

[simonlq1971@126.com](mailto:simonlq1971@126.com)
